# Cross-Language Validation and the Factor Structure of the Social-Emotional Competence Questionnaire for Pakistani Adolescents

**DOI:** 10.11621/pir.2023.0313

**Published:** 2023-09-30

**Authors:** Tahira Jabeen, Aneela Maqsood

**Affiliations:** a Fatima Jinnah Women University, Rawalpindi, Pakistan

**Keywords:** social-emotional competencies, reliability, validity, factor structure, psychometric characteristics, cross-language study

## Abstract

**Background:**

For the last few years, in the field of school education, social-emotional competencies have been gaining in importance because they result in positive attitudes and adaptation to school (Mella et al., 2021).

**Objective:**

The current study was designed to conduct Urdu translation, cross-language validation, and confirmatory factor analysis of the Social-Emotional Competence Questionnaire (SECQ) for Pakistani adolescents.

**Design:**

Urdu translation was done using the standard back-translation method. The data for the main study was collected using a non-random sampling method for 910 adolescents who were between 10 to 19 years old.

**Results:**

The findings of the pilot study (n = 64) show that the Social-Emotional Competence Questionnaire is a reliable questionnaire, as Cronbach’s alpha reliabilities of both versions (English and Urdu) were acceptable (Version 1 α = 0.77; Version 2 α = 0.77). After 15 days, the Pearson product-moment correlation was checked to meet the objective of cross-language validation, which resulted in a high correlation between the two forms (r = 0.88). The original author of the questionnaire had proposed a factor structure consisting of five factors, namely: self-awareness, social awareness, self-management, relationship management, and responsible decision-making. Results of the confirmatory factor analysis (CFA) in the present study also confirmed and supported the five-factor structure in comparison to other models for the Urdu version.

**Conclusion:**

The Social-Emotional Competence Questionnaire is a reliable and culturally-validated tool for adolescents attending school, which can be used in future research for measuring social-emotional competencies.

## Introduction

Social-emotional competencies are important for people of all ages, including young children, adolescents, and older adults. These skills particularly help children in managing problematic behaviors, thereby increasing interest and commitment toward their school. These are the basic advantages of these competencies. They also indirectly help to increase cooperation and problem-solving abilities among individuals, which improves family relationships and increases a healthy commitment towards society (Gokel & Dagli, 2017).

Since 1900, social-emotional competencies have been studied widely around the world, and intervention programs have been aimed at increasing these skills at various levels of education (Cristovao et al., 2020; Osher et al., 2016). These interventions are effective in improving skills of social-emotional learning, social behavior, and academic performance, as well as in decreasing problematic behaviors and psychological distress (Durlak et al., 2011). These skills are greatly influenced by culture, gender, and peer relationships. Males are less interested in, and less able to control, their emotions. So, males appear insensitive, non-cooperative, less emotional, and more aggressive (Chaplin & Aldao, 2013; Curby, et al, 2015). But females are more expressive than males, and can control their feelings in a constructive way, which enables them to better understand a variety of emotions, and their causes and consequences (Garner, et al, 2014).

As interest in the social-emotional domain has increased globally in the context of educational settings, this has increased the need to promote feelings of interpersonal skills of being accepted and recognized (Zhang et al., 2014). The social behavior of adolescents plays a vital role for students (Gómez-Ortiz et al., 2017), which favors school success (Cappadocia & Weiss, 2011).

It is widely-accepted that social competence is a comprehensive, empirical, and multi-faceted phenomena, which cannot be understood on its own, as it can include emotional regulation, prosocial behavior, the ability to adapt normatively, social adjustment, and perceived effectiveness in social interactions (Dirks et al., 2007; Losada, 2018; Santos et al., 2014). Gómez-Ortiz et al. (2017) state that social competence is the efficacy of social interaction, which stems from usage of socio-emotional skills in order to attain personal aims across diverse circumstances, and at different time periods. In this manner, social competence includes the cognitive and social-emotional capabilities of each individual to succeed under different circumstances, in forming healthy relationships among different people (Del Prette & Del Prette, 2005).

Hence, social competence means adjustment to changing demands in the school environment, in interpersonal relationships, emotional health, and acceptance among classmates (Losada et al., 2018). In addition, especially for adolescents, it is appropriate to study and evaluate interventions, to see their effect on social competence in educational settings (Gómez-Ortiz et al., 2017; Losada et al., 2017), because it is an important developmental period of maturation, and of subtle adaptation (as distinctive changes occur in this phase) in individuals (Bessa et al., 2019; Gómez-Ortiz et al., 2017).

The perception of what is appropriate social-emotional behavior changes from one context to another among different cultural groups (Kwong et al., 2018; Broekhuizen et al., 2017), and it is known as a unique aspect of childhood development. Country-level norms and values guide the appropriateness of social behavior and emotional expressivity (Wang & Zhang, 2020). When Asian people are communicating and interacting in public, they show limited expression of emotions and feelings. But group cohesiveness and feeling of belongingness are the most prominent features of some parts of Asian culture, i.e., East Asian culture (Liew & Zhou, 2022). If a focus is placed on research about social-emotional development, then evidence mostly pops up from regions such as Northern America, Europe, Great Britain, and Australia (Ren, et al., 2020; Ren & Pope Edwards, 2016).

For the past few years, those associated with the lives of children and adolescents, such as parents, teachers, researchers, and policy makers, have acknowledged the importance of social-emotional development for children and adolescents; this then has pushed people from Asia to study the cultural differences in social-emotional development (Chung et al., 2020). Even the most recent systematic review by Yong et al. (2023), found that most studies have come from the Asian continent – particularly from countries such as China and Hong Kong. Samples of these studies share Chinese ethnicity, which restricts their generalizability. ere is a scarcity of social-emotional development studies from Southeast, North, West, and Central Asia. Previous literature on social-emotional development indicated a clear need for scales to measure these competencies that are psychometrically rigorous and which have practical use in schools (Mantz et al., 2016).

## Objectives of the study

In the following paper, an effort was made to examine cross-language translation (to Urdu), and to carry out a confirmatory factor analysis of the Social-Emotional Competence Questionnaire, so that it can be used for evaluating the competencies among students, in light of the need for a reliable and valid questionnaire regarding social competencies in Pakistan. 

## Method

Through non-random sampling, adolescents who were currently enrolled in school were selected to take part in the study.

### Participants

The pilot study consisted of 33 males and 31 females of classes 9 or 10. Their age ranged from 13–17 years (M = 14.86 & SD = 0.97). The main phase of the study included 910 adolescents attending public schools, of classes 6 to 10. Of these, 454 were males, and 456 were females, with an age range 10 to 19 years (M = 13.45, SD = 1.63). If the students reported physical or psychological issues, or a history of traumatic events or surgery in the past six months, they were excluded from the study. In order to calculate a sample in any study for analyzing CFA, it is suggested to minimally include five to 10 individuals per item for that scale (Choudhry et al. 2018; Shuja et al., 2020).

### Parts of the study

The current study can be divided in two phases: the first phase in which the pilot study was based on Urdu translation and cross validation, and the second phase in which item-total correlation and various factor structures were computed as part of the main study.

### Process of Urdu Translation of the SECQ

For Urdu translation, the forward-backward method was used in the study (Anderson & Brislin, 1976; Hambleton, 1994), The details of each step of the translation process are given below.

- In Step 1, the English SECQ was given to five professionals to translate it into Urdu as accurately as possible, by keeping the construct of each item in mind. These professionals had a minimum of a Masters degree in different domains of the social sciences. They were experts in both the English and Urdu languages (Hambleton & Patsula, 1999). At the end of this phase, five independent Urdu translations were received by the researcher.
- In Step 2, the five translations were assembled up in a word.docx document. In order to check the Urdu translations, five different professionals were selected; their main task was to check the grammar, content, and construction of words and sentences. They were requested to propose new words or sentences if they found the content to be mistranslated, incorrect, or unclear, in order to obtain translations as close as possible to the meaning of the original version.
- In Step 3, five more professionals were hired who did not know about the questionnaire, to do five back-translations.
- In Step 4, the same five bilingual professionals as in Step 2, reviewed and evaluated the back translations, in order to obtain accurate and precise items for the final Urdu SECQ version.
- In Step 5, the final Urdu and English SECQ versions were administered to male and female students in two groups at two time periods. These students were bilinguals and they could understand both languages. After an interval of fifteen days, the same procedure was repeated with the same participants, in order to achieve cross-language validity for evaluating the structure and empirical equivalence for the Urdu and English SECQ.

### Procedure

This study was approved by the Ethical Committee of Fatima Jinnah Women University, Rawalpindi. After that approval, the SECQ author’s approval to use a questionnaire in the study was obtained. After the SECQ author’s approval, approval for data collection was obtained from the directorate of Federal Government Education Institutions. In the end, principals, school teachers, and students were informed about the study, its aim, significance, the right to withdraw at any time, and the matter of confidentiality. In total, the students spent 10 to 15 minutes completing the questionnaire, during which their questions were also addressed.

### Study Instrument

Individual descriptors for the students included age, gender, and their school class. The social competence of adolescents was assessed by the Social-Emotional Competence Questionnaire (SECQ).

### The Social-Emotional Competence Questionnaire (SECQ)

The Social-Emotional Competence Questionnaire (SECQ) was developed by Zhou and Ee in 2012. The SECQ is comprised of five subscales based on 25 items; these subscales are self-awareness, social awareness, self-management, relationship management, and responsible decision-making. The Collaborative for Academic, Social, and Emotional Learning (CASEL) grouped these competencies into *intrapersonal* (self-awareness, self-management), and *interpersonal* competencies (social awareness, relationship skills, and responsible decision-making) (Taylor et al., 2018). These social competencies are the means of adjustment to the changing demands of the school environment, interpersonal relationships, emotional health, and acceptance among classmates (Losada et al., 2018).

Questions of SECQ are scored on a six-point Likert scale which ranges from response options such as “not at all true of me” to “very true of me.” Overall, the authors reported reliability of 0.86 for the SECQ (Zhou & Ee, 2012). Resurrección et al. (2021) chose 221 primary education students, ages from 8 to 11 years, and for them they reported Cronbach’s alpha for each subscale: self-awareness = 0.64; social awareness = 0.72; self-management = 0.73; relationship management = 0.69; and responsible decision-making = 0.76. In the same way, Aguilar et al. (2019) selected 1494 participants from twelve schools with ages between 7 and 16 years old, and they reported reliability for the subscales of the SECQ as: self-awareness α = 0.72, social awareness α = 0.76, self-management α = 0.80, relationship management α = 0.72, and responsible decision-making α = 0.82. Sample items of the questionnaire are: *1)Iunderstand my moods and feelings; 2) If a friend is upset, I have a pretty good idea why;* and*. 3) I always try and comfort my friends when they are sad.*

## Results

Data was analyzed using the Statistical Package for Social Sciences (SPSS version 23, IBM Corp., Armonk, NY, USA), and the Analysis of Moment Structure (AMOS 22). In the first step, the mean, standard deviation, and alpha reliabilities of the English and Urdu versions, and the Pearson correlation, were calculated. In step 2, item-total correlation and different factor structures of the questionnaire were tested.

*Table 1* shows that alpha reliability coefficients were 0.77 for both forms, with correlation r = 0.88. This indicates that the Urdu SECQ had a good internal consistency and test-retest reliability, over the interval of 15 days in a small sample of 64 students.

**Table 1:** Pearson product-moment correlation coefficient, test-retest reliability of Urdu and English versions of the Social-Emotional Competence Questionnaire (SECQ) n = 64)

SECQ	Mean	Standard Deviation	Range	Cronbach Reliabilities	Correlation Coefficients	Significance Level
Urdu SECQ	89.17	11.49	53	0.77	0.88	0.000
English SECQ	88.28	11.67	55	0.77		

*Correlation is significant at 0.01 level (2 tailed)*

*Table 2* shows the corrected item-total correlation of the main study of the Social-Emotional Competence Questionnaire. Most of the items had an above .20 value for the corrected item-total correlation, which was acceptable (Abubakar et al., 2020; Piedmont, 2014).

**Table 2:** Corrected item-total correlation of Social-Emotional Competence Questionnaire (SECQ) (N = 910)

SECQ	Items	Item-Total Correlation
Self-Awareness	SAQ1	.571
	SAQ2	.575
	SAQ3	.564
	SAQ4	.589
	SAQ5	.524
Social Awareness	SOCAQ1	.558
	SOCAQ2	.482
	SOCAQ3	.463
	SOCAQ4	.484
	SOCAQ5	.554
Self-Management	SMQ1	.406
	SMQ2	.548
	SMQ3	.302
	SMQ4	.465
	SMQ5	.502
Relationship Management	RMQ1	.566
	RMQ2	.589
	RMQ3	.469
	RMQ4	.563
	RMQ5	.481
Responsible Decision Making	DMQ1	.637
	DMQ2	.670
	DMQ3	.654
	DMQ4	.625
	DMQ5	.634

**Figure 1.**
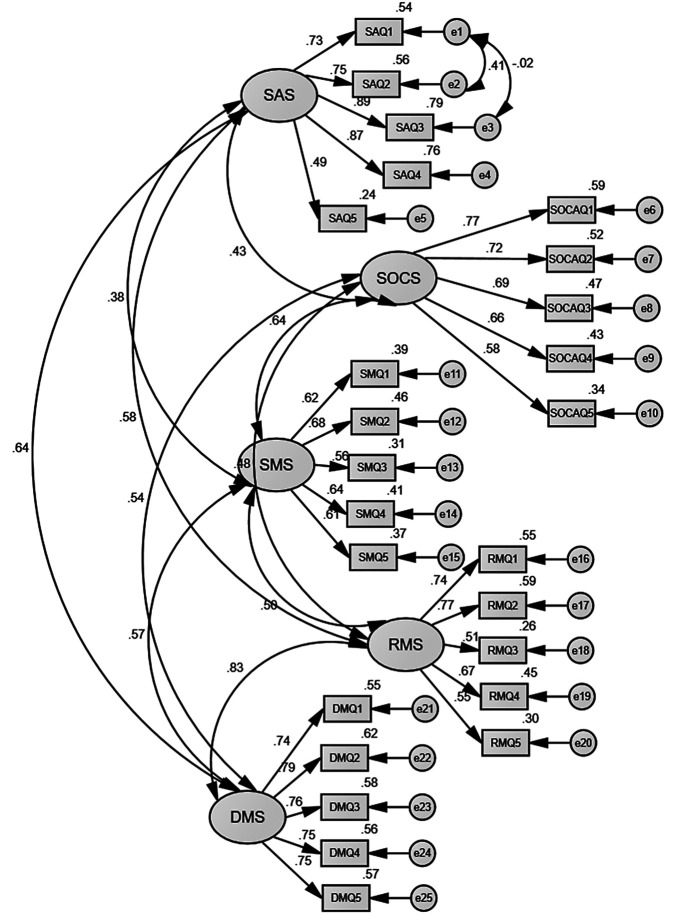
CFA five factors model for Social-Emotional Competence Questionnaire

**Figure 2.**
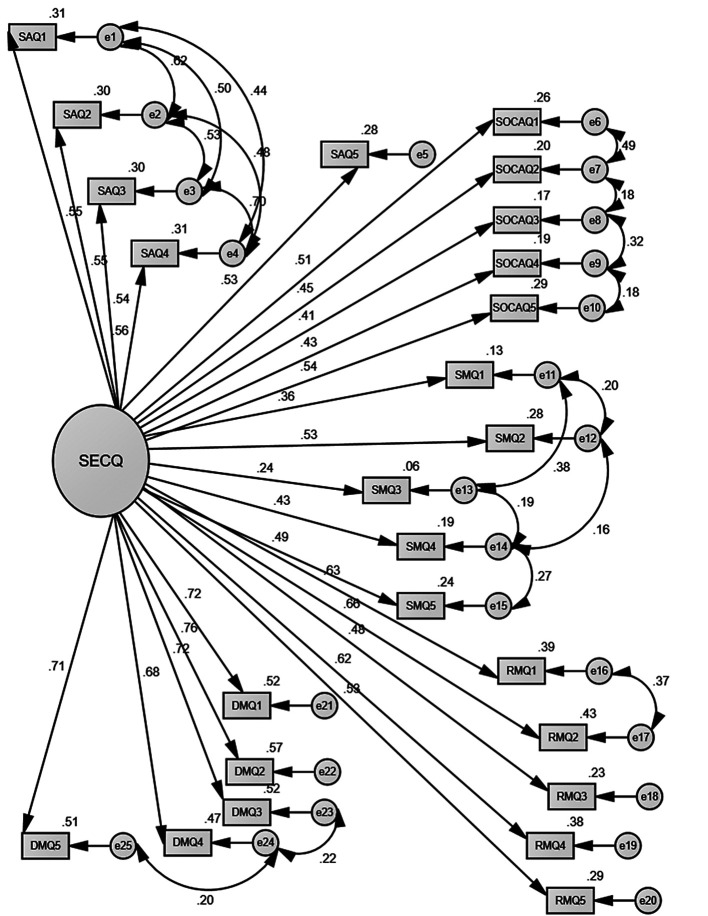
CFA model for uni-dimensional Social-Emotional Competence Questionnaire

**Figure 3.**
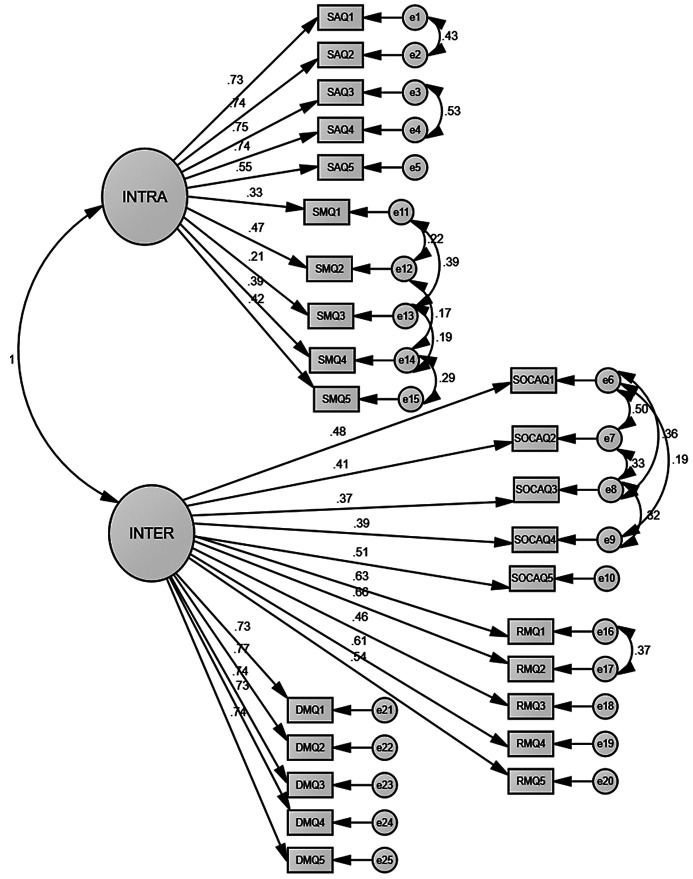
CFA model for two global factors Social-Emotional Competence Questionnaire

**Figure 4.**
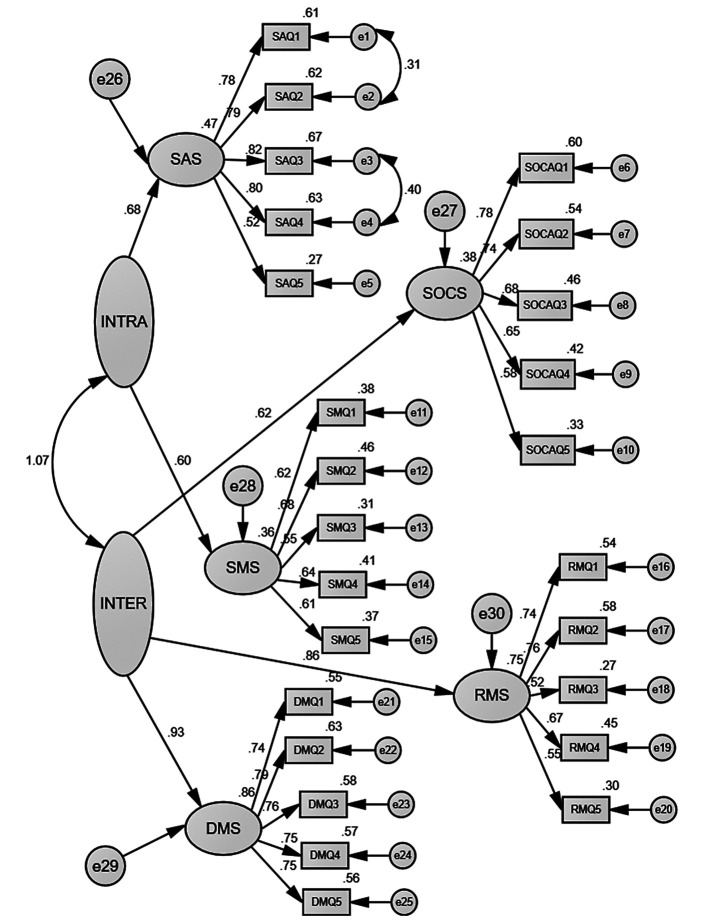
CFA model for five factors + two higher-order factors of Social-Emotional Competence Questionnaire

**Figure 5.**
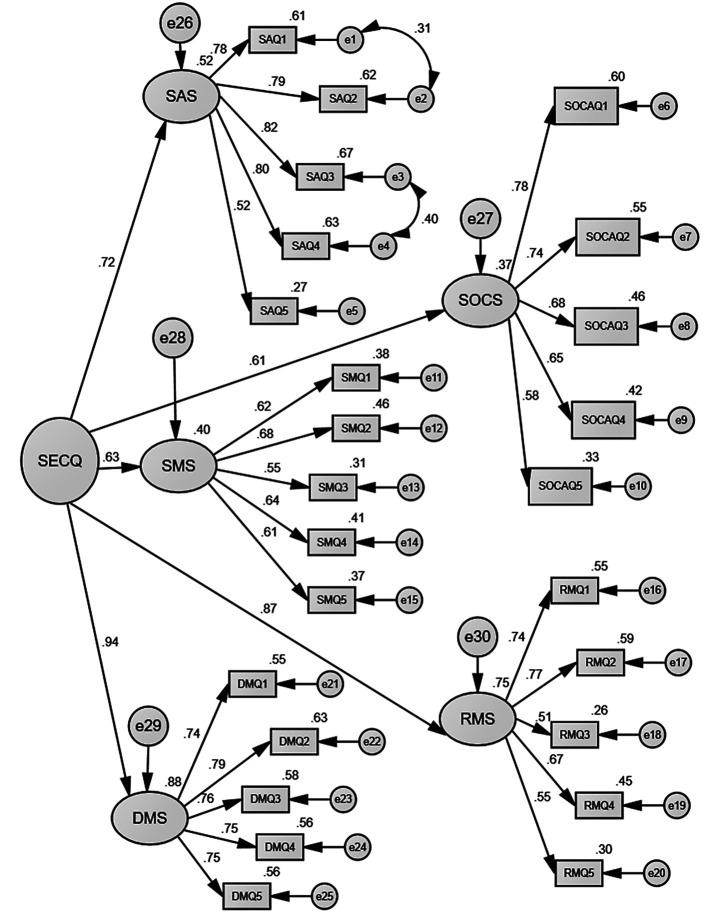
CFA model for five factors + one higher-order factor of Social-Emotional Competence Questionnaire

*Table 3* represents the variety of Model Fit Indices for the Urdu version of the Social-Emotional Competence Questionnaire. These models are: five factors, unidimensional, two global factors, five factors plus two, and one higher order factor. (Brown, 2015; Hooper et al., 2008).

**Table 3:** Model-fit Indexes for the Urdu Version of the Social-Emotional Competence Questionnaire (SECQ) (N=910)

	X^2^(df)	NFI	IFI	TLI	CFI	RMSEA
Five factors	1016.334 (263)	.904	.927	.917	.927	.056
Uni-dimensional	1252.518 (257)	.882	.904	.887	.903	.065
Two global factors	1470.249 (261)	.861	.883	.865	.883	.071
Five factors + two higher-order factor	1063.271 (267)	.900	.923	.913	.923	.057
Five factors + one higher-order factor	1067.551 (268)	.899	.923	.913	.922	.057

*Note. X^2^ = chi-square; df = degree of freedom; RMSEA = root mean square error of approximation; IFI = incremental fit index; NFI = normed fit index; CFI=comparative fit index; TLI =Tucker–Lewis index.*

## Discussion

The current study was conducted to determine the reliability, cross-language validity, and confirmatory factor analysis of the Social-Emotional Competence Questionnaire. It is one of the studies that aims to study the acceptability of fit and the inherent structure of the scale for the Pakistani population. Cross-language validation was done in a pilot study by administering both Urdu and English versions, with a 15-day interval in between, to 64 adolescent students. The results revealed that the Urdu and English versions of the questionnaire had a good level of reliability (Version 1 and Version 2 α = 0.77).

The findings of the present study regarding the reliability of the scale, are in line with most recently-conducted studies such as the ones by Kim (2021) and Portela-Pino et al. (2021), which have reported an average reliability of the SECQ as 0.88 and 0.91. Aguilar et al. (2019) and Resurrección et al. (2021) found a reliability of: self-awareness = 0.72, 0.64; social awareness = 0.76, 0.72; self-management = 0.80, 0.73; relationship management = 0.72, 0.69; and responsible decision-making = 0.82, 0.76, respectively.

After Cronbach’s alpha reliability met the criteria of satisfaction, then item-total correlation was computed, and the correlation value ranged from .30 to .67. These two values are same as previous studies (Abubakar et al., 2020; Piedmont, 2014). Apart from item-total correlation, factor loading of all items was checked, and all of these were higher than 0.30. All 25 items of the SECQ had a factor loading of a minimum of 0.30 and a maximum of 0.67, which had been recommended in a previous study (Jiménez, et al., 2018).

The present study also computed a factor structure based on five factors, uni-dimensional, two global factors, five factors + two higher order factors, and five factors + one higher order factor, of the Social-Emotional Competence Questionnaire. For testing acceptability of the proposed models, values of chi-square, degree of freedom, NFI, IFI, TLI, CFI, and RMSEA were checked. All proposed models of SECQ showed that the best-fit models for the scale such as chi-square and degree of freedom for five factor SECQ was 1016.334 (263), and the values of other indexes were: NFI = .904, IFI = .927, TLI = .917, CFI = .927, and RMSEA = .056.

The Chi-square and degree of freedom for uni-dimensional SECQ was 1252.518 (257), and the values of other indexes were: NFI = .882, IFI = .904, TLI = .887, CFI = .903, and RMSEA = .065. The Chi-square and degree of freedom for two global factor SECQ was 1470.249 (261), and the values of other indexes were: NFI = .861, IFI = .883, TLI = .865, CFI = .883, and RMSEA = .071. The Chi-square and degree of freedom for five factors plus two higher-order factor SECQ was 1063.271 (267). In addition, the values of other indexes were NFI=.900; IFI=.923; TLI=.913, CFI=.923 and RMSEA=.057. Lastly, the chi-square and degree of freedom for five factors plus one higher-order factor SECQ was 1067.551 (268), and the values of the other indexes were NFI = .899, IFI = .923, TLI = .913, CFI = .922, and RMSEA=.057.

Nearly all these five models of the SECQ supported the data efficiently. Among all these models, the five-factor model was the best-suited model for the SECQ, and this finding of CFA was the same as for the original study of the SECQ. In that study, authors initially indicated a marginal acceptable fit: X^2^ = 539.98 (df = 265, p<.001), X^2^/*df* = 2.04; RMSEA = .048; CFI = .89; and IFI = .89; but later on, for a sample of secondary school children, the CFA values of model fit were suitable: X^2^ = 712.20 (df = 265, p<.001), X^2^/df = 2.69; RMSEA = .069; CFI = .86; and IFI = .86 (Zhou & Ee, 2012).

In another study, Petric and Szamoskozi (2018) tested the five-factor structure on the sample of 546 in Hungary, in which results also showed a borderline acceptable fit of the CFA model as: X^2^ = 733.957 (df = 265, p<.001); X^2^/df = 2.77; RMSEA = .056; CFI = .89; and IFI = .89.

The latest study of the SECQ by Dinh et al. (2021) tested the number of CFAs for the SECQ such as five factors, two global factor, five factors plus two and one higher order factor. Out of these four models, three showed a fitness of acceptable, except two global factors such as normed X^2^ on five factors: CFI = 2.43, RMSEA = .901, SRMR = .034, AIC = 99977.209, and BIC = 100410.227; normed X^2^ on five factors + one higher-order factor: CFI = 2.42, RMSEA = .901, SRMR = .039, AIC = 99974.101, and BIC = 100387.681; normed X^2^ on five factors + two higher-order factor: CFI = 2.43, RMSEA = .900, SRMR = .039, AIC = 99978.752, BIC=100391.355; and normed X^2^ on the two global factor model: CFI = 4.11, RMSEA = .779, SRMR = .050, AIC = 100583.624, and BIC = 100973.572.

The findings of present study are the same as the aforementioned study, except for the two global factor SECQ models, which were not supported by Vietnamese culture. This also supports the notion that the culture of each country is unique, and can cause changes in the expression of social competence among its people (Kwong et al., 2018; Broekhuizen et al., 2017). The results of the present research are in line with the predefined objectives that are supported by previous research. In the recent years, the SECQ has been translated and culturally-validated for residents of Hungary and Vietnam.

The findings of the present study further provided evidence of cross-language validity from Pakistan by testing a variety of SECQ models. Practitioners who are working with adolescents might use the validated SECQ instrument in both clinical and non-clinical settings. The SECQ is a short, straightforward, and easily comprehensible questionnaire, which provides an opportunity to administer it more than one time to evaluate the level of social competence of adolescents. Future studies can also use other measures with the SECQ in order to establish convergent and discriminant validity among different scales. By applying the standards of evidence-based practice, the psychometric qualities of the SECQ can be explored in other Asian countries.

## Conclusion

Among models of the SECQ such as the five-factor model, uni-dimensional, two global factor, five factors plus two and one higher order factors, the five-factor model emerged as a promising factor, and the SECQ was culturally-validated scale for determining social competence on the sample of adolescent students in Pakistan. Hence, future researchers should use the mean of the five-dimensions model for social competence.
